# Short-term sedimentation dynamics in mesotidal marshes

**DOI:** 10.1038/s41598-022-26708-8

**Published:** 2023-02-02

**Authors:** A. Rita Carrasco, Katerina Kombiadou, Ana Matias

**Affiliations:** Centre for Marine and Environmental Research (CIMA), University of Algarve, Campus of Gambelas, 8005-139, Faro, Portugal

**Keywords:** Wetlands ecology, Hydrology

## Abstract

One of the key questions about wetlands resilience to sea-level rise is whether sediment supply will be enough to keep them coping with growing inundation levels. To address this question, researchers have put a lot of effort into field data collection and ecogeomorphic modelling, in an attempt to identify the tipping points of marsh survival. This study uses fieldwork data to characterize the sediment fluxes between the tidal flats and salt marshes, in the Ria Formosa lagoon (Portugal). Sediment fluxes were measured from the tidal channel towards the mid-upper marsh, during neap and spring tide conditions. The flow magnitude was measured, and induced transport was determined based on shear velocities. Deposition rates, instantaneous suspended sediment and near-bed velocities were linked through theoretical formulas and used to characterize time-averaged conditions for sediment delivery and deposition to the site. The results showed that suspended sediment concentrations and sediment deposition varied across the transect with no specific relation to elevation. Maximum water depths were recorded in the vegetated tidal flat, and the maximum currents were flood dominated, in the order of 0.20 m/s, in the low marsh due to flow-plant interactions and an increase of turbulence. Deposition rates ranged between 20 to 45 g/m^2^/hr, after a complete tidal cycle, and were higher in the mid-upper marsh. Hydroperiod was not the main contributor to sediment deposition in the study area. Sediment transport was tidally driven, strongly two-dimension during the cycle, and highly influenced by the vegetation. Measurements of marsh sediment flux obtained in our work are diverse from the ones found in the literature and evidence the importance of considering spatio-temporal variability of vegetated platforms in assessing overall marsh bed level changes.

## Introduction

Salt marshes are valuable ecosystems of great ecological, geomorphological, economic, and social importance^1–3^. The accumulation of inorganic and organic sediments allows salt marshes to keep in pace with sea-level rise up to a given threshold rate^4–7^ and to eventually reach a biogeomorphic equilibrium^8^. The processes controlling the exchange of sediment with the bed, including settling velocities and related deposition/entrainment thresholds, are highly complex and variable. Factors such as timing, frequency and height of inundation^9–11^, distance to the sediment source^12,13^, and seasonal variations in water levels and wind regime^14,15^ were found to affect sediment deposition. Not least, the halophytic vegetation colonizing salt marshes also contributes to marsh vertical accretion by enhancing mineral deposition, through the capture of sediment particles (e.g.^16^) and reduction of turbulence kinetic energy (e.g.^17–19^), as well as organic sedimentation due to root growth and organic litter deposition (e.g.^20,21^).

Variations in canopy morphology and the physical structure of individual plants itself control fine-scale hydrodynamics, and influence particle advection and settling^22^, reducing marsh erosion^13,14,23^. An increase in marsh biomass can increase the effective settling velocity of particles in suspension over the marsh surface through turbulent kinetic energy dissipation^18^. Even though the velocity attenuation can enhance local sediment retention, it can reduce sediment supply downdrift^24^. Aside from flow conditions, fine particle flocculation and settling depend on a variety of local factors, including suspended concentrations and organic content. On the other hand, rather than settling, the biological trapping of sediment on the leaves and stems of plants can be the main factor inducing deposition of fine grained sediments in marsh environments^5^. While the consequences of canopy height have been studied extensively, the mechanisms by which the salt marsh vegetation modifies the hydrodynamics and influences the sediment dynamics are still uncertain. Descriptions in the literature on the role of canopy in streamflow turbulence are quite diverse, suggesting that there is no standard typical pattern of attenuation (or enhancement), varying at small spatial scales, and being dependent on local ecological and hydrogeomorphic interactions.

Several studies have investigated the interactions between vegetation and marsh bed elevation, although the majority of these studies have focused on only one habitat (e.g. focused on marsh habitats spanning a salinity gradient^25^; focused on *Spartina alterniflora*^18^, or focused on *spartina sp.*^20^), disregarding the sediment transference within the marsh zonation. Both^26,27^ provide interesting perspectives on quantifying bed changes in salt marsh compartments, however, without describing extensive measurements of transported material across marsh succession. Likewise, observational studies provide limited insights into how much of the sediment delivered is actually retained on the tidal flat and marsh platform surfaces across the wetlands. Vegetation sedimentation feedbacks are only one of many potentially important interactions occurring at salt marsh platforms^26,28^, and a variety of methods have been developed for measuring and monitoring surface dynamics in tidal wetlands (see reviews in^28,29^). The most commonly employed methods to determine suspended sediment concentration are collecting water samples at varying locations^30^, and deploying bed deposition traps across marsh succession (as described in^13^). The suspended sediment concentration determines the amount of sediment that can potentially be deposited on a marsh^31^ and often varies both at large scales (i.e., between marshes) and within a single marsh (e.g.^11,28,30^). The sediment deposition (or retention) rate is estimated near the bed, presents high spatial variability, and is dependent on the tidal range and wind-wave conditions (e.g.^13^), and on the presence of intertidal vegetation^3,32^. Estimates of suspended sediment concentrations and deposition rates described in literature vary at small spatial scales, and commonly refer to spring tide conditions, likely corresponding to peak sediment transfer conditions (see examples of suspended sediment concentrations and deposition rates from literature in Tables S1 and S2). This highlights the need for fieldwork based studies that build a more comprehensive picture of marsh sedimentation dynamics from neap to spring tide cycles.

Many studies have been carried out in the last decade to assess the rates of sediment transport and deposition on tidal flats and salt marshes, however, a need to characterize the transport fluxes between the various habitats as a function of tidal range, their position relative to mean sea level, and flow asymmetries in the vegetation effect remain. The present study provides new insights on sediment transport at a sediment restricted wetland, over spring and neap tide cycles, by identifying the sediment transport and deposition drivers. The field site extends over ca. 110 m of a salt marsh and vegetated tidal flat platform. The tidal ranges covered are representative of the maximum and minimum sediment input to the area, and the obtained findings are relevant to attest local marsh vulnerability and stability. The results contribute to understanding the relationship between inorganic deposition and biophysical drivers (i.e., habitat type, elevation, hydroperiod, and currents) in natural wetlands, and demonstrate the importance of considering the small spatial variations in sediment transport studies.

## Methods

No plants were collected or harmed during this study, and all research involving plants followed relevant national, and international guidelines and legislation.

### Study area

**Figure 1:**
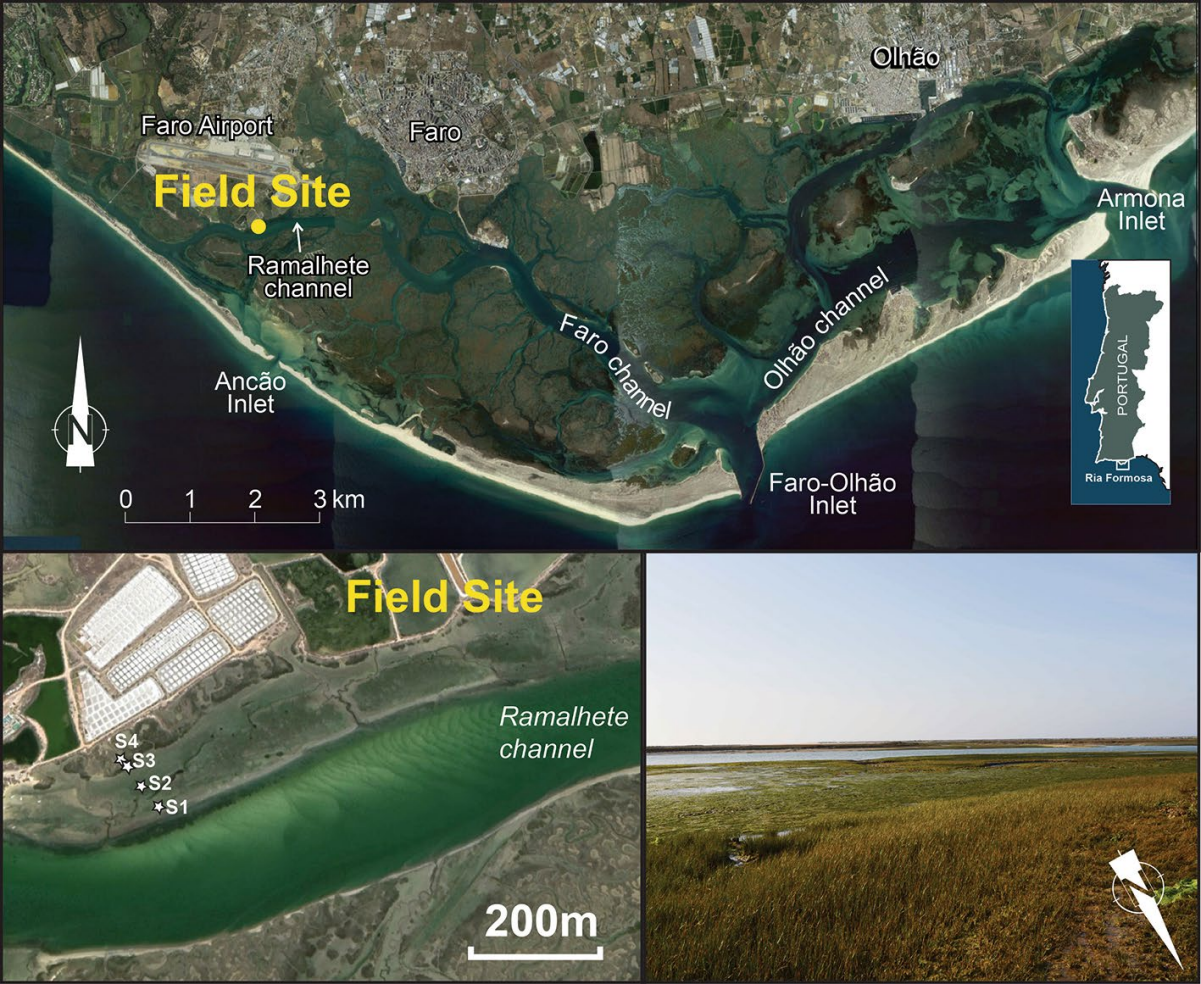
Location of the field site in the Ria Formosa lagoon western sector over a satellite image collected in 2019 (South Portugal; upper panel); zoom to monitoring stations S1 to S4 (left lower panel); and field view of the studied site (right lower panel). Map generated with ArcGIS 10.8 (http://www.esri.com) and Adobe Illustrator 2022. Map data: Google Earth 7.3, image Landsat / Copernicus.

The study site encloses a wetland area bordering Ramalhete Channel, in the western part of the Ria Formosa lagoon, a mesotidal system located in southern Portugal (Fig. 1). Lunar tides are semi-diurnal, with a mean tidal range of about 2 m that can reach up to 3.5 m during spring tides. Offshore waves have no major propagation inside the lagoon^33,34^. Water circulation inside the lagoon is mostly driven by tides. The lagoon extends over 55 km along the coast and is connected to the ocean through six tidal inlets^35^. The three westmost inlets of the system (Ancão, Faro-Olhão, and Armona), which together capture ca. 90% of the total prism, are highly interconnected, with a strong residual circulation from Faro-Olhão Inlet directed towards Ancão and Armona inlets (located in Fig. 1), during both spring and neap tides^36^. The tidal currents in Ramalhete Channel, connecting the Faro-Olhão and Ancão Inlet, have high tidal asymmetry and shifts in tidal dominance, from flood to ebb. There are no significant fluvial inputs into the lagoon, with a yearly average terrestrial sediment influx of around 2 × 10^5^ m^3^/yr^37^, reaching the system through small streams. The main sediment delivery to the system is through the inlets, though there are few studies assessing related fluxes. The net sediment entry through the stabilized Faro-Olhão Inlet is estimated at 1.4 × 10^5^ m^3^/year^38^. Recent sedimentation rates in the marsh of the westmost edge of the lagoon were estimated at 1.1 ± 0.1 mm/yr^39^.

Th﻿e﻿﻿ lagoon system is composed of large salt marsh patches, tidal flats and a complex net of natural, and partially dredged tidal channels. The tidal flats (vegetated and non-vegetated) and salt marshes represent more than 2/3 of the total lagoon area. The salt marshes comprise silt and fine sand^40^, while coarser (sand to shingle) shell-rich sediment, of marine provenance, is found on tidal channels and the lower domain of intertidal flats^41^. The dominant intertidal species are *Spartina maritima* and the seagrass *Zostera noltei*, the latter occupying an estimated area of 1304 ha, which represent 45% of the total intertidal area^42^.

### Experimental setup and data analysis

An experimental setup was deployed in the study area to assess dominant local topography, hydrodynamics (water levels and current velocities), Suspended Sediment Concentrations (SSCs), Deposition Rates (DRs), vegetation characteristics, and bed sediment grain size and organic matter content. Measurements were made during a full tide cycle, on a spring tide (tidal range = 3.2 m), and on a neap tide (tidal range = 1.8 m). Sampling was conducted in four wetland stations: S1 and S2 in a vegetated tidal flat comprising *Zostera noltei*; S3 in the low marsh comprising *Spartina maritima*; and S4 in the mid-upper marsh with the most abundant species of *Sarcocornia perennis* and *Atriplex portucaloides* (see S1 to S4, Fig. 1); the tidal flat is interrupted by a small oblique secondary tidal creek that flows near S2 station.

**Figure 2:**
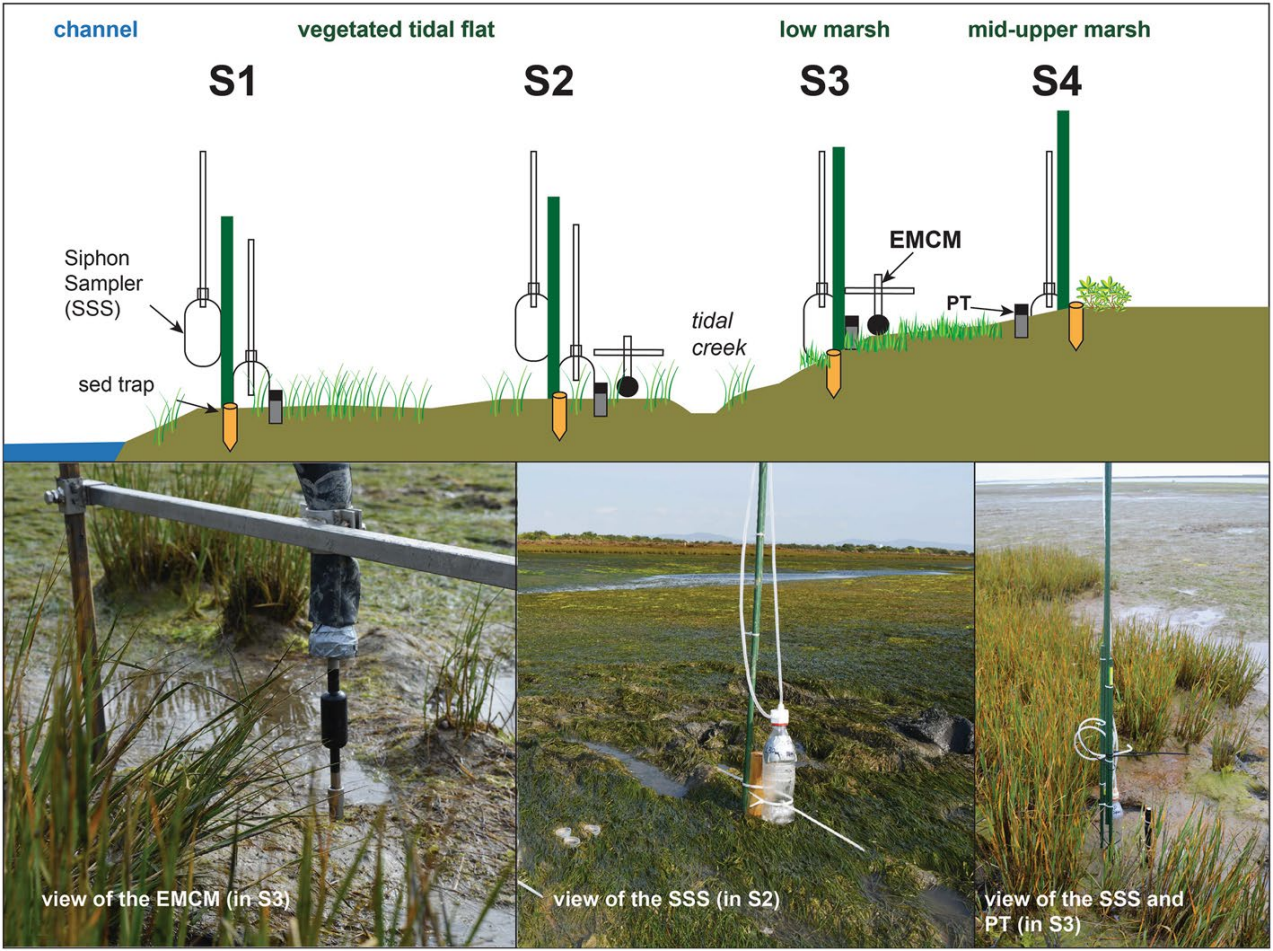
Deployment of the sediment traps, SSSs and devices (electromagnetic current meter EMCM; pressure transducer PT) in the stations (S1 to S4) during spring tide (sketch is exaggerated in the vertical).

Stations of sediment sampling and equipment deployment along the transect are illustrated in Fig. 2. During neap tide there was no data collection in S4, since the inundation time of the station was very short. The profile elevation was measured using Real Time Kinematic Differential Global Positioning System (RTK-DGPS, Trimble R6; vertical error in the order of few centimetres), and the slope of each habitat within a transect was calculated and expressed in percentage (%). Vegetation at each point was characterized by the canopy height, calculated as the average shoot length.

Suspended Sediment Samplers (SSSs) were installed during low tide in the monitored stations using siphon samplers (Fig. 2) and recovered in the next low tide. These samplers consist of 0.5 L bottles with two holes on the cap, one for water intake and the other for air exhaust, according to the method described in^13^. Each intake tube is adjusted to form a siphon (i.e., inverse U), allowing to control the water level at which intake starts. Siphons were aligned at the same elevation along the transect for spring and neap tides, which means that all SSSs were collecting at the same time within the tidal cycle. During spring tide, in S1 and S2 at the tidal flat, SSSs were sampling at 0.1, 0.9, and 1.2 m from the bed, while at S3 SSSs were sampling at 0.7 and 1.0 m from the bed, and at S4 the SSS was sampling at 0.1 m from the bed (Fig. 2). During neap tide, in S1 and S2, SSSs were sampling at 0.1 and 0.9 m from the bed, while at S3 the SSS was sampling at 0.7 m from the bed.

Surficial sediment samples were collected in each habitat to characterize the sediment grain size (d_50_) and content of organic matter (% OM). Sediment traps were installed in 3 replicates, during low tide, at each sampling point to measure the short-term sediment deposition rate (i.e., deposition over a tidal cycle, following procedures of^43^). Traps consisted of 3 cm diameter pre-labeled cylindrical tubes (Falcon® tubes, 50 ml). Traps and sediment samples were transported to the laboratory and maintained in a fridge. The sediment content was washed, and both the inorganic and organic weights were determined.

The measured inorganic DR (g/m^2^/hr) was calculated as:

1 $${\text{DR}} = {\raise0.7ex\hbox{${{\text{W}}_{{{\text{DS}}}} }$} \!\mathord{\left/ {\vphantom {{{\text{W}}_{{{\text{DS}}}} } {{\text{A}} \cdot {\text{T}}}}}\right.\kern-0pt} \!\lower0.7ex\hbox{${{\text{A}} \cdot {\text{T}}}$}}$$

where *W*_DS_ is the weight of deposited sediment (in grams), *A* is the area of the sediment trap opening (m^2^), and *T* is in hours. Two different tide durations were considered to compute DRs, one assuming *T* equal to the hydroperiod in each station, and one assuming *T* equal to the entire tide duration (~ 12.4 h). These measured DRs are hereon mentioned as flood and tide DRs (DR_flood_ and DR_tide_, respectively). The former is an expression of the actual deposition rate within the flood phase, during the period in which each station is inundated (and therefore active deposition can take place). The latter is the value used to compare with DRs in literature, which typically corresponds to values averaged over multiple tidal cycles (thus accounting for the entire tide duration).

Tide levels were measured in the field using pressure sensors (PT, InSitu Inc. Level TROLL; ~ 2 cm from the bed), deployed from S2 towards S4 (Fig. 2). Velocity currents were measured at 20 cm from the bed, using an electromagnetic current meter (EMCM; Infinity Series JFE Advantech Co., Ltd; in S2 to S4; Fig. 2), and raw data (recording interval: 30 s) were filtered using a 10 min moving average for cross-shore and longshore components. To identify tidal asymmetry and assess the related phase dominance, tidal current skewness was calculated through the formula described in^44^ by which:

2 $$Sk_{U} = \frac{{\frac{1}{N - 1}\mathop \sum \nolimits_{t = 1}^{N} \left( {U_{t} - \overline{U}} \right)^{3} }}{{\left( {\frac{1}{N - 1}\mathop \sum \nolimits_{t = 1}^{N} \left( {U_{t} - \overline{U}} \right)^{2} } \right)^{{{\raise0.7ex\hbox{$3$} \!\mathord{\left/ {\vphantom {3 2}}\right.\kern-0pt} \!\lower0.7ex\hbox{$2$}}}} }}$$

where N is the number of recordings, *U*_*t*_ is the input velocity signal and $\overline{U}$ is the mean velocity. Positive/negative skewness indicates flood/ebb dominance (assuming that flood currents are positive).

Complementary to the measured DRs, theoretical DRs were also determined from the data, allowing us to link the sediment and flow data collected, and validate the deposition patterns from the traps. The theoretical deposition rate was determined based on^45^ formula:

3 $${\text{DR}} = \left\{ {\begin{array}{*{20}c} {{\text{C}}_{{\text{b}}} \cdot {\text{w}}_{{\text{s}}} \cdot \left( {1 - \frac{{{\uptau }_{{\text{b}}} }}{{{\uptau }_{{{\text{cd}}}} }}} \right)} & {{\uptau }_{{\text{b}}} < {\uptau }_{{{\text{cd}}}} } \\ 0 & {{\uptau }_{{\text{b}}} \ge {\uptau }_{{{\text{cd}}}} } \\ \end{array} } \right.$$

where C_b_ is the SSC at the bed, w_s_ is the flock settling velocity, τ_b_ is the bed shear stress and τ_cd_ is the corresponding critical value for deposition.

To determine the settling rate of the flocculates, the modified Stokes’ velocity for cohesive sediment was used, taking shape factors α and β (α = β = 1 for perfectly spherical particles):

4 $${\text{w}}_{{\text{s}}} = \frac{{\upalpha }}{{\upbeta }} \cdot \frac{{\left( {{\uprho }_{{\text{s}}} - {\uprho }_{{\text{w}}} } \right) \cdot {\text{g}} \cdot {\text{D}}_{50}^{2} }}{{{\uprho }_{{\text{w}}} \cdot 18 \cdot {\upnu }}}$$

where ρ_w_ and ρ_s_ are the densities of the water and sediment, respectively and ν is the kinematic viscosity of water (~ 10^6^ m^2^/s).

The bed shear stress τ_b_ was calculated from the measured current magnitude, |*U*| using the law of the wall:

5 $$\begin{array}{*{20}c} \\ {{\uptau }_{{\text{b}}} = {\uprho }_{{\text{w}}} \cdot {\text{u}}_{*}^{2} , {\text{u}}_{*} = \frac{\left| U \right| \cdot \kappa }{{\ln \left( {{\raise0.7ex\hbox{$z$} \!\mathord{\left/ {\vphantom {z {z_{0} }}}\right.\kern-0pt} \!\lower0.7ex\hbox{${z_{0} }$}}} \right)}} } \\ \end{array} { }$$

where *κ* is the von Kármán constant (~ 0.4) and *z*_0_ is the roughness length. For *Zostera noltei*, the roughness length was estimated at 5 mm^46^, value that was also used in the other stations, in lack of related estimate for marsh plants.

The critical shear for deposition, τ_cd_, was calculated using the formula^47^:

6 $$\sqrt {\frac{{{\uptau }_{{{\text{cd}}}} }}{{{\uprho }_{{\text{w}}} }}} = \left\{ {\begin{array}{*{20}c} {0.008} & {{\text{w}}_{{\text{s}}} \le 5 \cdot 10^{ - 5} {\text{m}}/{\text{s}}} \\ {0.094 + 0.02 \cdot {\text{log}}_{10} \left( {{\text{w}}_{{\text{s}}} } \right)} & {3 \cdot 10^{ - 4} \le {\text{w}}_{{\text{s}}} \le 5 \cdot 10^{ - 5} {\text{m}}/{\text{s}}} \\ {0.023} & {{\text{w}}_{{\text{s}}} \ge 3 \cdot 10^{ - 4} {\text{m}}/{\text{s}}} \\ \end{array} } \right.$$

Theoretical values of minimum SSCs needed for these DRs were also calculated, assuming that there is constant deposition (i.e., setting τ_b_ = 0), and compared with the field results.

## Results

The studied morphologies are all vegetated, and the physical characteristics are presented in Table 1 (and illustrated in Figure S1 in supplementary material). Grain size (d_50_) increased from the tidal flat towards the mid-upper marsh, while the hydroperiod varied inversely with bed elevation. The organic matter was quite variable between habitats and between spring and neap tide, and no clear pattern can be highlighted. Lower OM % in S2 (Table 1) might be explained by some fine sediment deposition driven by the shallow tidal creek (see Figure S1B in supplementary material). Canopy height varied between habitats, with higher stem heights for the upper marsh than for the seagrass leaves (Table 1).

**Table 1:** Ecogeomorphic characteristics of the monitored stations.

Station	S1	S2	S3	S4
Habitat (*Dominant plant species*)	Tidal flat (*Z. noltei*)	Tidal flat (*Z. noltei*)	Low marsh (*S. maritima*)	Mid-upper marsh (*Sarcocornia perennis and Atriplex portucaloides*)
Average of stem height (cm)	20.00	15.00	35.00	40.00
Elevation with respect to mean sea level (MSL, m)	0.20	0.20	0	0.60
Slope (%) between consecutive environments	0.90	2.50	8.00	
Mean hydroperiod (hours)	7.40	7.40	6.70	4.21
d_50_ (μm);	16.68	17.10	19.30	18.20
OM (%) on the deposited sediment for spring tide	41.72	23.48	31.76	34.90

### Hydroperiod and current velocities

**Figure 3:**
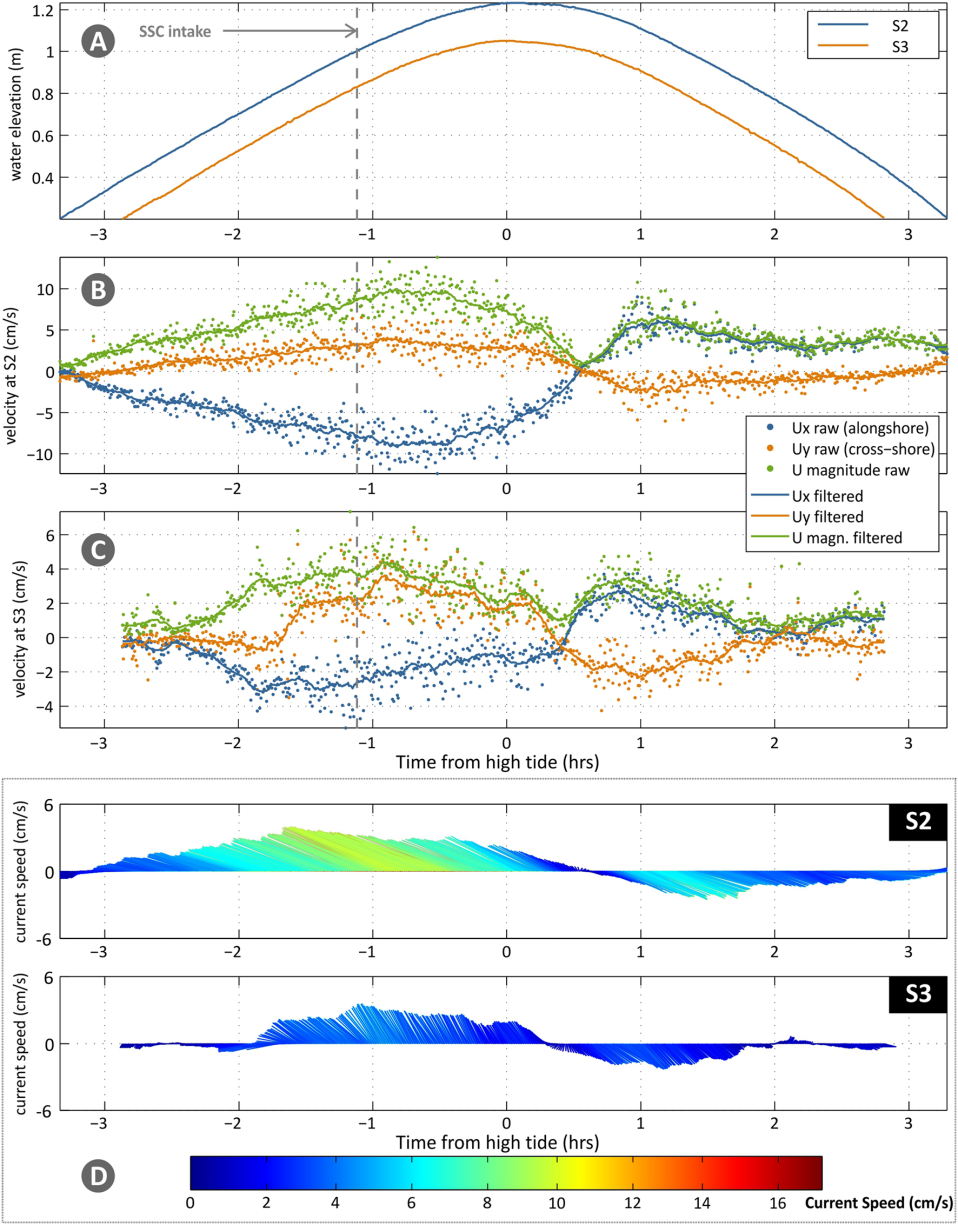
Water level ((**A**), for all stations; in m) and current velocities along the transect ((**B**) and (**C**) for S2 and S3; in cm/s) for neap tide conditions. (**B**) and (**C**) show alongshore (blue colour; positive values denote east direction) and cross-shore components (orange colour; positive values denote landward north direction), as magnitude (green colour) and as raw (points) and filtered (lines) data. (**D**) shows vector timeseries (filtered data), where line colour denotes speed (reference to colour-bar; same scale for neap and spring tides); the length of the arrows refers to the current speed (in the y-axis).

No strong wind episodes were experienced on the sampling days, or other unusual meteorological conditions. Maximum water depths estimated in *Zostera noltei* meadows ranged from 1.23 to 1.76 m, and for *Spartina maritima*, from 1.05 to 1.56 m (referred to Mean Sea Level). Under both neap and spring tide conditions, currents had a major alongshore component in the tidal flat that was reduced further inland (see the abrupt change in mean direction between S3 and S4, Table S2), with the cross-shore component becoming leading at S4 (~ 1.4 cm/s, Table S2). S4 recorded the lowest velocities which can be explained not only by the distance from the channel and bed elevation but also caused by the effect of plant’s height and density (Table 1). An increase in current velocities was noted at the boundary of the low marsh during spring tide conditions (from 0.41 cm/s to 5.01 cm/s, Table 1 and Fig. 3), due to an increase in turbulence, related to the fast change in slope and the transition from the flat to the low marsh vegetation (Figure S1C in supplementary material).

**Figure 4:**
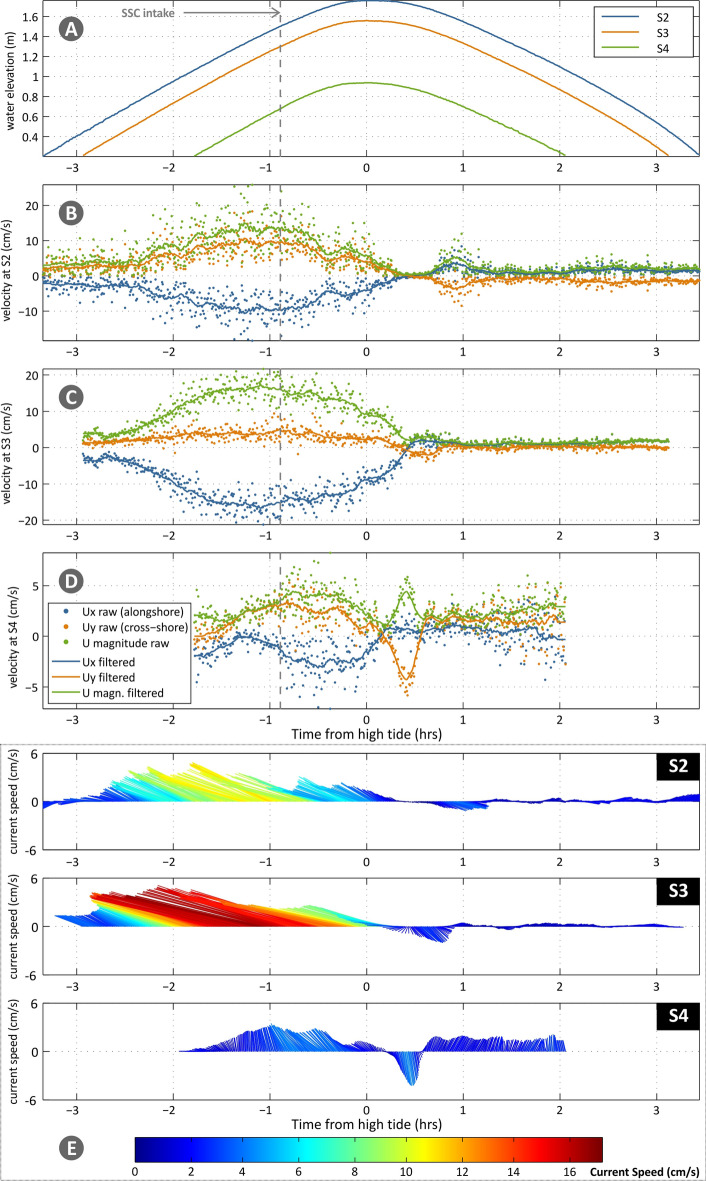
Water level ((**A**), for all stations; in m) and current velocities along the transect ((**B**), (**C**) and (D) for S2, S3 and S4; in cm/s) for spring tide conditions. (**B**), (**C**) and (**D**) show alongshore (blue colour; positive values denote east direction) and cross-shore components (orange colour; positive values denote landward north direction), as magnitude (green colour) and as raw (points) and filtered (lines) data. (**E**) shows vector timeseries (filtered data), where line colour denotes speed (reference to colourbar; same scale for neap and spring tides); the length of the arrows refers to the current speed (red in the y-axis).

The recorded timeseries of velocities (including raw and filtered current velocities and vector timeseries) are given in Fig. 3 for neap tide and in Fig. 4 for spring tide. A lag of around 0.4 h is noted for all conditions between hightide and current reversal (high water slack) in the field. During neap tide, the currents at S2 (Fig. 3B) had a significant alongshore component and, as they propagated to S3 during the flood phase, they turned more cross-shore (Fig. 3C). The current attenuation from S2 to S3 was of the order of 50% at peak current speed (Fig. 3D). The strong steering of the flow was also present during the ebb phase, with an increase of the alongshore ebb current component from S3 to S2 (Fig. 3C). During spring tide (Fig. 4), the flood currents in S2 showed higher variability in direction (angled at 60–76° to the transect, Fig. 4E), however, magnitudes were not strongly enhanced, compared to neap tide conditions (maximum increase of 25%). Contrastingly, very high acceleration of the flow was observed in S3 (Fig. 4C), both with respect to flood flow along the transect (transition from S2 to S3), as well as compared to neap tide flood phase in the same station (Fig. 3C). The direction of the flow did not significantly change between S2 and S3 (Fig. 4E). Ebb flow during spring tide is surprisingly lower than the neap tide. One hour into the ebb cycle, the velocities in S2 and S3 reduce to near zero, while in S4 the flow reverses (turning shoreward); these changes remain for the rest of the recording period. It is noted that comparing the filtered and raw data for S4 (Fig. 4D), the intense scatter of the latter (with no clear trend and variability of cross-shore component between − 3.5 and + 4 cm/s) likely indicates highly turbulent flow. Thus, the positive filtered cross-shore component in the station during ebb could be related to intense turbulent fluctuations and not an actual shoreward directed flow during ebbing tide. We, thus, consider that the velocity in S4 should also be near-zero (as noted for the other 2 stations).

The intense relaxation of the ebb flow during spring tide both in terms of the related flood phase and compared to the recorded ebb during neap tide is likely related to topography and canalisation of the flow over the non frequently inundated part of the marsh, which, can often produce a complex velocity field^48^.

**Figure 5:**
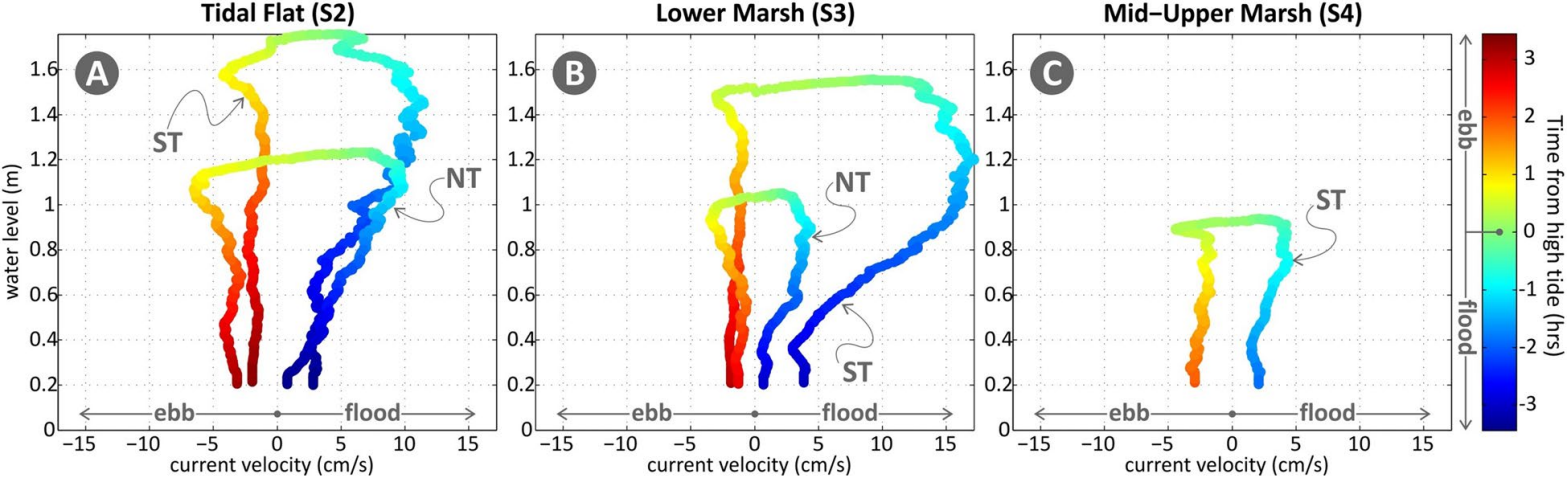
Water level versus flow velocity for stations S2 (**A**), S3 (**B**) and S4 (**C**) and for neap and spring tides (NT, ST). The colour-scale (explained in the bar) is uniform for both tides, with negative values (blue to cyan) corresponding to flood and positive ones (yellow to red) to ebb phases.

Examining the variability of current speed with water elevation (Fig. 5) during neap and spring tidal cycles, the overall flood dominance in current velocities (as also shown by the skewness values in Table S2), especially prominent during spring tide in stations S2 and S3, is easily noted (Fig. 5). Therefore, it can be expected that the influx (and potentially the trapping) of sediment is significantly higher than the flushing out during the ebb phase. The increase in current speeds from S2 to S3 during spring tide is most likely due to boundary effects at the marsh edge (S3), which appear significantly less prominent under neap tide conditions. The variability in S3 for the neap tide is very similar to the one of S4 for the spring tide, both associated with similar inundation levels. This likely points to similar flow-plant (*Spartina* and *Sarcocornia*) interactions in the two stations.

### Spatial sediment transport and deposition

**Figure 6:**
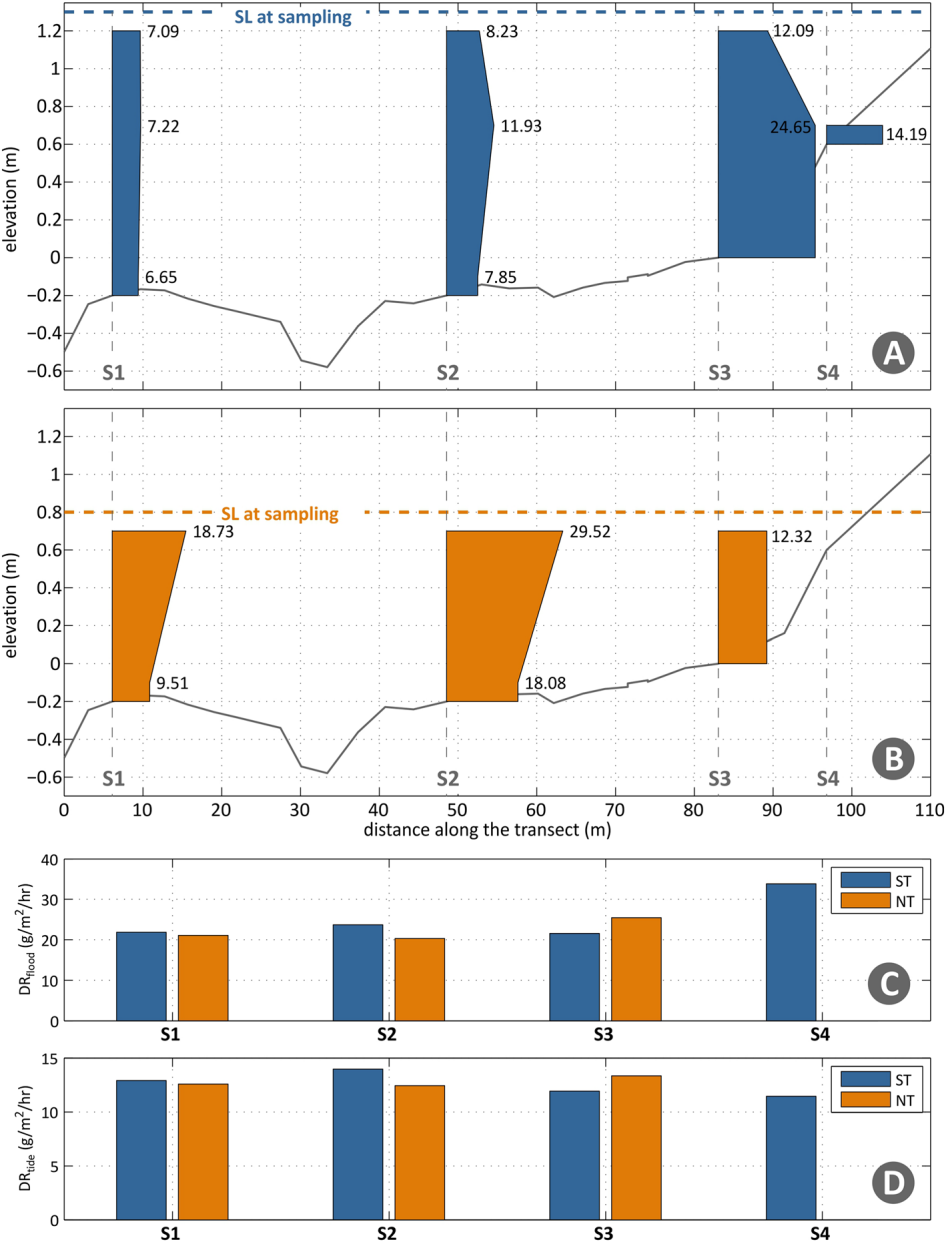
(**A**) Recorded SSCs during spring tide (mg/l); (**B**) Recorded SSCs during neap tide (mg/l); and (**C**) and (**D**) DR_flood_ and DR_tide_ (g/m^2^/hr), in blue and orange, respectively, during both spring (ST) and neap tides (NT); SL–sea-level.

Suspended sediment concentrations are higher during the neap tide, when compared with the spring tide (Fig. 6A and B). During spring tide conditions, SSCs increase from the tidal flat towards the low marsh (from S2 to S3, Fig. 6A), with the opposite trend observed under neap tide (Fig. 6B). While for spring tide the SSCs in the tidal flat, above the bed and near the water surface were of the same magnitude, in the low marsh, SSCs are higher near the bed canopy (~ 24.6 mg/l measured for the low marsh, Fig. 6A). During neap tide, SSCs are always higher near the water surface (~ 29.5 mg/l measured for S2, Fig. 6B), in accordance with the log variability of the current with depth and higher potential sediment flux at the surface. Calculated flood deposition rates (DR_flood_) were higher for the spring tide in the mid-upper marsh and increased with bed elevation (Fig. 6C). The small increase in flood DRs between S1 and S2 in spring tide could reflect the local effect of the oblique shallow tidal creek. Considering the tide DR values (DR_tide_), a decrease with station elevation is noted for spring tide, with deposition rates ranging from 14 gr/m^2^/hr in S1 to 11.4 gr/m^2^/hr in S4. Very similar values are noted for the neap tide, with low variability along the transect, ranging between 12.5 to 13.3 gr/m^2^/hr.

**Figure 7:**
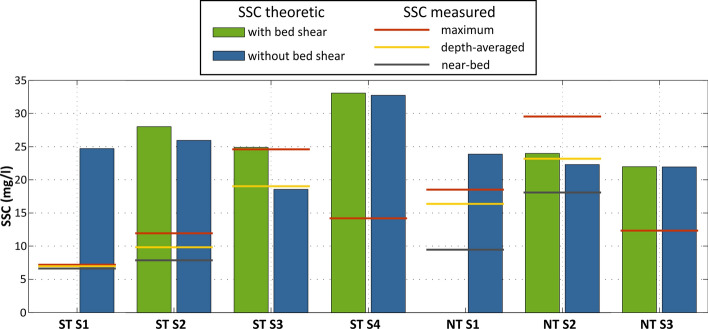
Comparison of SSC (mg/l) values: theoretic time-averaged, calculated from the flood DR (DR_flood_; Fig. 6C) values (bars: values with and without bed shear stress calculation are shown; only the latter is shown for S1, due to lack of flow measurements) and instantaneous, field measured (lines: maximum for the station, depth-averaged and near-bed concentrations are given as red, yellow and grey lines); for S3 and S4 some of the lines are overlapped due to few SSSs in the column.

The theoretical time-averaged SSCs, calculated from the measured flood DRs and the sediment setting velocities (Eq. (1)) are given in Fig. 7 (see formulas description in the methods section). Calculations were made both assessing the bed shear stress conditions and disregarding them (τb = 0), thus assuming constant deposition during the hydroperiod of each station. The latter corresponds to the minimum average SSCs needed to produce the measured flood DRs (Fig. 6C). For station S1 only values assuming constant deposition are shown, due to missing flow data. Measured SSCs are also given in the plot, as maximum and depth-averaged values in the column and as values near the bed (in some cases, i.e., S3 and S4, values overlap due to few SSSs in the column or overlapping measurements for spring tide in S1). It can be noted that, overall, the instantaneous SSCs measured are lower than the corresponding theoretic ones, especially for spring tide conditions, where even maximum measured SSCs are 2 times lower. In S3, for the neap tide, the measurement is also 2 times lower than the theoretic value. Taking into account that measured SSCs are instantaneous and actual values during the flood are expected to fluctuate significantly, (a) it is not surprising that the measured values do not reflect the temporal variability and/or sediment pulses (i.e., local resuspension, sediment influx from creeks) and (b) these theoretical values can serve as an indirect measure of time-averaged SSCs in the field, during a full tidal cycle.

## Discussion

This study measured the suspended sediment concentrations (SSCs), deposition (DRs), and related hydrodynamic patterns in a mesotidal marsh in Ria Formosa lagoon, for neap and spring tide conditions. The recorded current velocity profiles, over a tidal cycle, presented significant speed differences among habitats, and between tidal ranges, being not inversely related to the distance from open water, as observed in similar studies^19^. The flow speed, under both tidal regimes, was highly skewed and flood-dominated, while at the transition from the tidal flat towards the marsh, strong topographic steering was identified (Fig. 5). Neap tidal currents enter the domain at a high angle (alongshore, from the east) to the transect at the tidal flat (station nearest to the channel), gradually turning more cross-shore (~ 45° towards inland) at the edge of the marsh (Fig. 3). The flow attenuation at the transition from the tidal flat to the low marsh was around 50%. Conversely, during spring tide, flood currents accelerated by up to 25% in the same area, while the current direction remained stable (Fig. 4). This is probably related to the transitional flow near the low marsh edge, where the higher (compared to neap tide) spring tidal currents are expected to become significantly more turbulent at the transition from the flexible *Zostera* seagrass meadow to the more rigid *Spartina* vegetation^49^. A similar pattern of peak velocities and turbulent kinetic energy identified at the edge of a *Spartina* patch decreasing values with the distance from the vegetation edge was observed by^10^, when the water flow could not move above the canopy but is forced through the canopy. Ebb flow was near-zero throughout the transect, likely due to complex drainage flows along the upper, non-frequently inundated marsh platform (Fig. 4). Overall, the variability in the velocity profiles could be related to the unsteadiness of tidal currents (influenced by the topography), the change in water depth from spring to neap tides, and the heterogeneity of the vegetation canopy^14^.

Net sediment transport in the transect was not fully cross-shore, as observed in other studies describing sediment transport fluxes in wetlands (Fig. 6; e.g.^13^). Measured SSCs showed high variability with tide level and along the transect (Fig. 6A and B), ranging from 7–24 mg/l for spring tide to 10–29 mg/l for neap conditions, values that are one order of magnitude lower than the SSCs reported for macrotidal and larger mesotidal estuaries (e.g.^50^, and Table S1), but close to the median SSCs determined by^51^, in de other of ~ 30 mg/l. Though we haven’t verified higher SSCs for spring tide conditions, as observed by^51^. Our SSCs patterns are not spatially consistent with the results of many other published studies (e.g.^30,31^), describing an inverse relationship between suspended load and elevation (from the tidal flat towards the upper marsh). However, being instantaneous measurements, our SSC data are difficult to compare with other sites. Indeed, the recorded instantaneous SSCs measured are often lower than the corresponding theoretic ones, especially for spring tide conditions, (Fig. 7). However, theoretical values, that reliably express the bulk sediment settling during the full tidal cycle, can serve as an indirect measure of time-averaged SSCs.

A decrease in sediment concentrations from the bed towards the water surface, during spring tide was observed (Fig. 6A), explained by the dominant tidal current orientation as demonstrated in the cases reported in^29,52^. Similar to findings by^53^, this study demonstrates increased sediment deposition (DRs) along the transect toward the upper marsh surface (Fig. 6) under spring tide conditions, probably linked to the influx of SSCs through a tidal creek (due to an increase in tidal range). The measured deposition rates represent the effective net deposition-resuspension rate, i.e., the deposited material, as well as resuspended material^32^, varying with elevation and habitat. In this study, measured tide DRs (DR_tide_) ranged between 12.5–14 and 12–13.3 g/m^2^/hr, for the *Zostera* and *Spartina*, respectively (Fig. 6C)^54^, whereas in a nearby location (around 450 m downdrift), measured DRs of 7–14 g/tide in *Zostera* and 1–5 g/tide in *Spartina*. The observed differences in the magnitude of deposited sediment can be explained by the spatial variability at short scales and by differences in the data collection approaches. Compared with our results, maximum velocities recorded by^54^ were low, roughly < 10 cm/s, representative of a weaker current regime, in varying topography. SSCs from the same study (3–15 mg/l; measured using OBSs for 2–3 days at various locations near the Ramalhete Channel bank) were also significantly lower than the instantaneous measurements from the SSSs in our site (6.7–29.5 mg/l). These disparities point to distinct mass influx, transport and deposition regimes in the two areas, albeit their close proximity (ca. 450 m apart). This very strong variability in the spatial controls at small scales, suggests that isolated measurements of sediment transport can hardly be used as representative of an entire system.

The recorded tide DRs (DR_tide_) in this coastal lagoon area (ca. 12.5 g/m^2^/hr) are one-fourth of the ones measured by^13^ for the extensive marshes in the Rattekaai Estuary (ca. 53 g/m^2^/hr, Table S1 in supplementary material), but closer to the^31^ estimates (around 22 g/m^2^/hr, Table S1 in supplementary material). We observed no significant difference between the measured DRs, neither along the transect nor between spring and neap tides, despite the varying SSCs values. Sediment deposition appears slightly enhanced in the low marsh for neap tides (Fig. 6D), while a small gradual reduction in deposition from the main channel can be noted, as observed in other wetlands systems^29^. Generally, lower elevation within the tidal frame and closer proximity to the source of tidal inundation result in higher sedimentation rates^6,25,29^. The closer proximity to the main channel increases the flood duration, and increases the time for sediment deposition to occur^6,54,55^. The high values of tide DRs towards the mid-upper marsh, despite the lower hydroperiod, can be explained by an increased stem height and branching level^56^. Stem density has been shown to be an important factor influencing sediment deposition^14^, with nonlinear trapping effects^57–59^. Results from^54^ showed that the flow interaction with the bed in vegetated marshes depends mostly on the vegetation density at each level of the canopy, and supports the current observations. The site specific patterns of transport and tidal flow (flood dominance, high alongshore component near the channel with topographic steering along the transect) deviate from known patterns and highlight the importance of conducting field experiments to complement current knowledge, as well as for calibrating numerical models. Importantly our results can be used to assess sediment budget and marsh edge instability^27^ in fetch-limited marshes, and provides useful a outlook for wetlands restoration. We highlight the need to understand small-scale factors to reveal the role of biophysical parameters in the transition between the tidal flat and salt marsh, where the inundation period is not determinant of short-term bed level changes. One of the key strengths of using short-term deposition measurements is the ability to identify and quantify the composition of fine-scale inputs, difficult to identify with medium-term studies (such as the one undertaken by^28^, or such as SSC and DR computations over weeks^51^. Still, SSCs can be highly variable, even though more accurate than continuous loggers like OBSs, the latter indirect methods to assess sediment concentrations, that might not be representative of SSCs within the full flood cycle. DRs on the other hand (determined using multiple traps) can provide a better idea of the sedimentation in the domain, though potentially remobilized material along the transect cannot be separated from newly imported matter within the sample^43^.

The short-term sampling and DR estimates conducted allowed to establish the net deposition over a single tidal cycle reasonably well, and minimising the error associated with vertical sediment pulses (i.e., resuspension or strong wind episodes) that is more likely to occur when measuring during consecutive tides. Besides, the short-term sampling employed allowed to perform a comparative analysis of sediment fluxes across a wetland platform, between spring and neap tide conditions. The study could be extended with SSCs sampling at different moments in the tide and across a two-dimension functional grid, which is likely important due to the spatio-temporal variability in the current directions identified. Given the scarcity of field data on wetlands sediment transport, the collected dataset is useful to further analysis in flow-turbulence changes by a *Spartina* vegetation, and for calibrating and validating hydrodynamic and sediment transport models for the study area.

## Conclusions

The obtained results provide insights on the dynamics and variability of flow and mass transfer along a transition from the vegetated tidal flat to the upper marsh, showing that: a) the tidal flow along the transect is strongly two-dimensional with a high degree of topographic steering and shifting direction under both spring and neap flood cycles; b) flood-dominated tidal dynamics was identified, with very low ebbing velocities, especially during spring tide conditions, allowing to concur that sediment out-fluxes are likely negligible and to characterize the site as a sediment sink; c) instantaneous SSCs were mostly lower than the corresponding theoretical estimates based on the sedimentation along the profile, pointing to a high temporal variability in the sediment influx during the flood phase to the area; d) deposition rates were relatively high, especially compared to previous measurements in a neighboring patch, indicating the high spatial variability in sediment fluxes and deposition; e) no significant differences were identified in the deposition rates between neap and spring tides along the tidal flat up to the low marsh; f) even though the influence of plants was not directly assessed, the high sediment retention in the area and changes in flow along the transect (i.e., the increased velocity at the low marsh edge under spring tides) indicate that vegetation likely contributed to attenuate flow and to increase sediments trapping, though improved measurements of plant density and biomass are needed.

Measurements of marsh sediment flux obtained in our work are diverse from the ones found in the literature, considering salt marsh in other geomorphological settings, but also within the same lagoon system. The work highlights the need for further fieldwork based studies that build a more comprehensive picture of the complex salt marsh dynamics in confined tidal lagoons, where spatial morphological variability can generate complex flow dynamics. Further experiments of short-term deposition and medium-term accretion across a broader range of sites in the Ria Formosa lagoon, and modelling of vegetation influence are thus still required.

## Supplementary Information

Supplementary Information.

## Data Availability

The datasets generated during and/or analyzed during the current study are available from the corresponding author on reasonable request.
