# Supplementary material for: Short-term sedimentation dynamics in mesotidal marshes
Source: Sci Rep. 2023 Feb 2;13:1921. doi: 10.1038/s41598-022-26708-8 (PMC9895031; doi:10.1038/s41598-022-26708-8)
Supplement: Supplementary file 1 — Supplementary Information. [file 41598_2022_26708_MOESM1_ESM.docx]

**Short-term sedimentation dynamics in mesotidal marshes**

A. Rita Carrasco^(1)*^, Katerina Kombiadou^(1)^, Ana Matias^(1)^

^(1)^ Centre for Marine and Environmental Research (CIMA), University of Algarve, Campus of Gambelas, Faro, Portugal, *corresponding author: [azarcos@ualg.pt](mailto:azarcos@ualg.pt)

# Supplementary material

Table S1. Selection of Suspended Sediment Concentration (SSC) and Deposition Rate (DR) measurements in meso- and macrotidal marshes.

| Authors | Location | Environment | DR* | SSC (mg/l) | Observations  *Vegetation* |
| --- | --- | --- | --- | --- | --- |
| ^1^ | Ramalhete Channel, Ria Formosa, Portugal | Mid-low marsh | 7-14 g/m^2^/tide | - | Spring and neap tides |
|  |  |  |  |  | *Spartina maritima* |
|  |  | Tidal flat | 1-5 g/m^2^/tide | - | Spring and neap tides |
|  |  |  |  |  | *Zostera Noltei* |
|  |  | Channel bank | - | 3-15 | Average over 2-3 days |
|  |  |  |  |  | *non-vegetated* |
| ^2^ | St. Jones River, Delaware, USA | Low marsh | 22.3 g/m^2^/h | 10 to 1900 | Spring tides |
|  |  |  |  |  | *Spartina alterniflora* |
|  |  | Upper marsh | 3.6  g/m^2^/h | - | Spring tides |
|  |  |  |  |  | *Phragmites australis* |
| ^3^ | Kingsport marsh, Minas Basin, Canada | Low marsh | 30.9±17.7 g/m^2^/tide | 106±66 | Average of 62 tides |
|  |  |  |  |  | *Spartina alterniflora* |
|  |  | Marsh edge | 15.5±5.9 g/m^2^/tide | 53±24 | Average of 62 tides |
|  |  |  |  |  | *Spartina patens* |
|  |  | Upper edge | 15.3±6.1 g/m^2^/tide | - | Average of 62 tides |
|  |  |  |  |  | *Spartina patens* |
| ^4^ | Rattekaai marsh, the Netherlands | Low marsh | 53.0±11.0 g/m^2^/h | 576 ± 137 (averaged across transect) | Spring tides |
|  |  |  |  |  | *Spartina anglica, Halimione portulacoides, Elymus athericus* and *Puccinellia maritima* |
|  |  | Upper marsh | 31.4±8.7 g/m^2^/h |  | Spring tides |
|  |  |  |  |  | *Spartina anglica, Halimione portulacoides, Elymus athericus* and *Puccinellia maritima* |
|  | Sint Annaland, the Netherlands | Upper marsh | 7.3±17.5 g/m^2^/h | 34 ± 5 (averaged across transect) | Spring tides |
|  |  |  |  |  | *Halimione portulacoides, Elymus athericus* and *Festuca rubra* |
| ^5^ | Phillips creek, Hog Island Bay, USA | Upper marsh | - | 31 | Spring tides |
|  |  |  |  |  | *Spartina Alterniflora* |
| ^6^ | Paulina marsh, the Netherlands | Upper marsh | - | 50 | Spring- neap tides cycle |
|  |  |  |  |  | *Puccinellia Towsendii, Aster Tripolium, Atriplex Portulacoides,* and *Elytrigia Pungens* |
| ^7^ | Wanggang, China | Upper marsh | - | 340 | Average over the year |
|  |  |  |  |  | *Suaeda salsa, Spartina angelica, and Spartina alterniflora* |
| ^8^ | Blackwater River, Chesapeake Bay, USA | Low marsh | - | 54 | Measured during flood |
|  |  |  |  |  | *Spartina patens* and *Schoenoplectus americanus* |
|  |  |  |  |  |  |
| ^9^ | Europe and North America | Tidal marshes** | - | [10-500] | not available |

* Please note that units of DRs vary for the several studies, either g/m^2^/h or g/m^2^/tide.

** no specific reference to upper or low marsh.


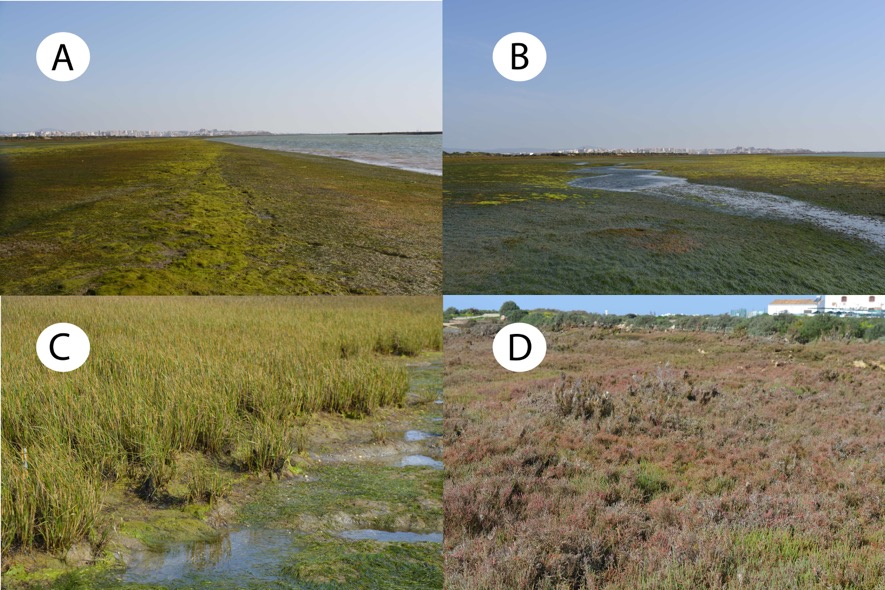


Figure S1. Illustrations of the studied salt-marsh vegetation. (A) *Zostera noltei* near Ramalhete channel; (B) view of the tidal creek cutting the tidal flat; (C) pioneer *Spartina maritima*; and (D) dominant *Sarcocornia perennis* in the mid-upper marsh.

Table S2. Maximum water depths, near-bed current velocities and tidal asymmetry derived by the skewness of the tidal currents (calculated from eq. (2)).

|  | Neap tide | | | Spring tide | | |
| --- | --- | --- | --- | --- | --- | --- |
| Parameter | S2 | S3 | S4* | S2 | S3 | S4 |
| Maximum  water depth (m) | 1.23 | 1.05 | - | 1.76 | 1.56 | 0.94 |
| Mean alongshore velocity (cm/s) | 1.57 | 0.41 | - | 2.32 | 5.02 | 0.49 |
| Mean cross-shore velocity (cm/s) | 0.77 | 0.33 | - | 1.11 | 1.52 | 1.38 |
| Current skewness [dominance] | 0.21 [flood] | 0.11 [flood] | - | 0.54 [flood] | 0.36 [flood] | 0.19 [flood] |

^*not reached during neap tide^

**References for supplementary material**

1. Neumeier, U. & Ciavola, P. Flow Resistance and Associated Sedimentary Processes in a *Spartina maritima* Salt-Marsh. *J Coast Res* **20**, 435–447 (2004).

2. Moskalski, S. M. & Sommerfield, C. K. Suspended sediment deposition and trapping efficiency in a Delaware salt marsh. *Geomorphology* **139–140**, 195–204 (2012).

3. Poirier, E., van Proosdij, D. & Milligan, T. G. The effect of source suspended sediment concentration on the sediment dynamics of a macrotidal creek and salt marsh. *Cont Shelf Res* **148**, 130–138 (2017).

4. Ma, Z., Ysebaert, T., van der Wal, D. & Herman, P. M. J. Conditional effects of tides and waves on short-term marsh sedimentation dynamics. *Earth Surf Process Landf* **43**, 2243–2255 (2018).

5. Christiansen, T., Wiberg, P. L. & Milligan, T. G. Flow and sediment transport on a tidal salt marsh surface. *Estuar Coast Shelf Sci* **50**, 315–331 (2000).

6. Temmerman, S., Govers, G., Wartel, S. & Meire, P. Spatial and temporal factors controlling short-term sedimentation in a salt and freshwater tidal marsh, scheldt estuary, Belgium, SW Netherlands. *Earth Surf Process Landf* **28**, 739–755 (2003).

7. Wang, A. A simplified conception model for salt marsh sedimentation rate calculation: Response to changing suspended sediment concentration-A case study of Wanggang salt marsh, Jiangsu Province, China. *Frontiers of Earth Science in China* **4**, 403–409 (2010).

8. Ganju, N. K. *et al.* Sediment transport-based metrics of wetland stability. *Geophysical Research Letters* **42**, 7992–8000 (2015).

9. Coleman, D. J. *et al.* Reconciling models and measurements of marsh vulnerability to sea level rise. *Limnol Oceanogr Lett* **7**, 140–149 (2022).
